# Supplementary figures and images for: Susceptibility to Zika virus in a Collaborative Cross mouse strain is induced by Irf3 deficiency in vitro but requires other variants in vivo
Source: PLoS Pathog. 2023 Sep 21;19(9):e1011446. doi: 10.1371/journal.ppat.1011446 (PMC10547207; doi:10.1371/journal.ppat.1011446)

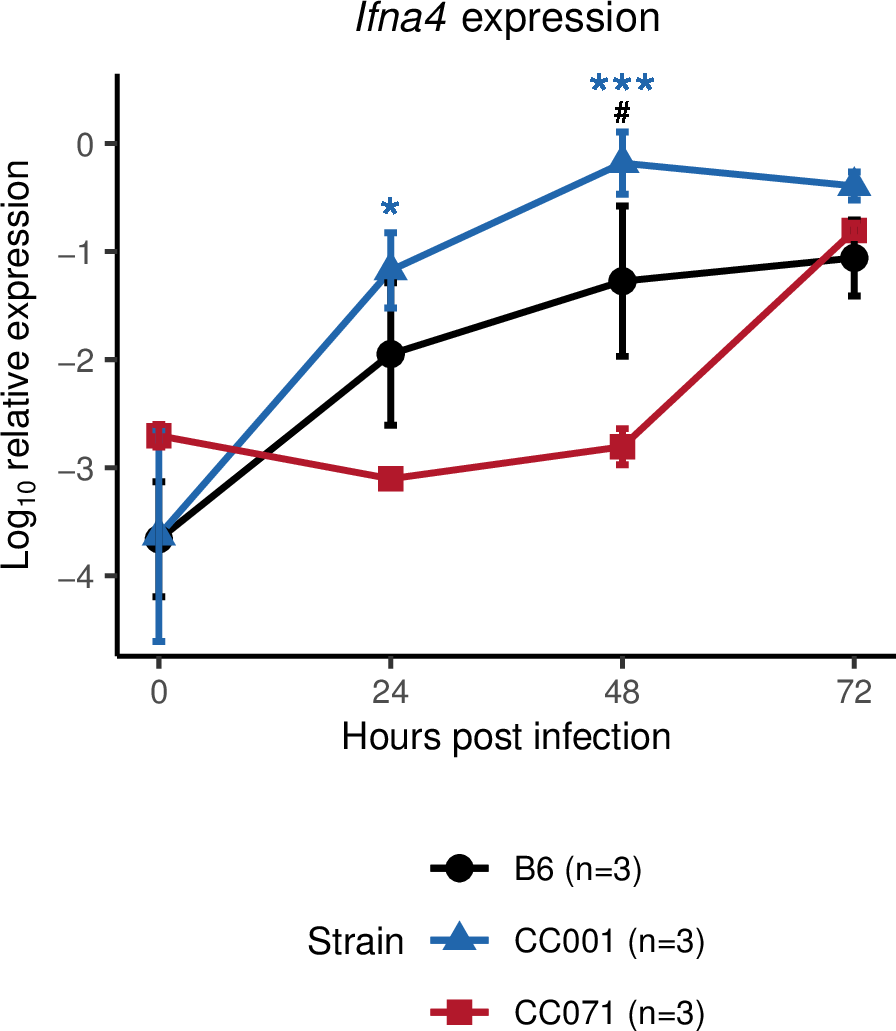

Supplement: S1 Fig — MEFs derived from B6 (gray circles), CC001 (blue triangles) and CC071 (red squares) were infected with ZIKV at a MOI of 5. Ifna4 expression was determined by RT-qPCR on MEFs total RNA by normalizing to Tbp housekeeping gene. Data are mean +/- sem from 3 biological replicates. For one CC001, one B6 and one CC071 replicates at 0 hpi and one CC071 replicate at 24 hpi, gene expression was below the limit of detection. Blue asterisks and black hashes show statistical significance of CC071 compared to CC001 and to B6, respectively (ANOVA followed by post-hoc Tukey HSD, */# p < 0.05, *** p < 0.001). (TIF) [file ppat.1011446.s001.tif]

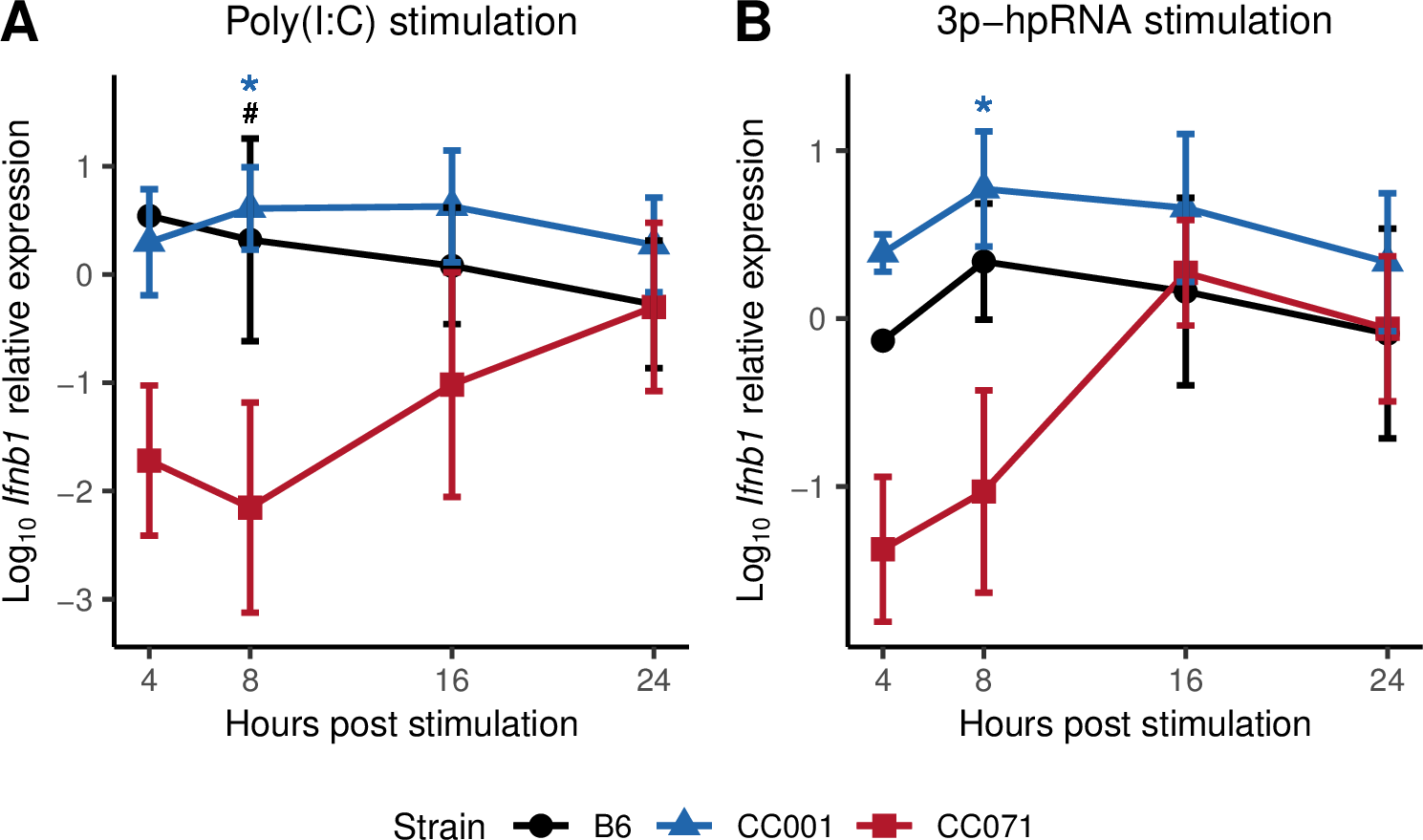

Supplement: S2 Fig — MEFs were transfected with either (A) poly(I:C), which activates both TLR and RIG-I pathways, or (B) 3p-hpRNA, a RIG-I agonist. Ifnb1 expression was determined as in Fig 1B. Data are mean +/- sem from 3 biological replicates for CC001 and CC071 (2 at 4 hours) or 2 biological replicates for B6 (1 at 4 hours). Blue asterisks and black hashes show statistical significance of CC071 compared to CC001 and to B6, respectively (ANOVA followed by post-hoc Tukey HSD, */# p < 0.05). (TIF) [file ppat.1011446.s002.tif]

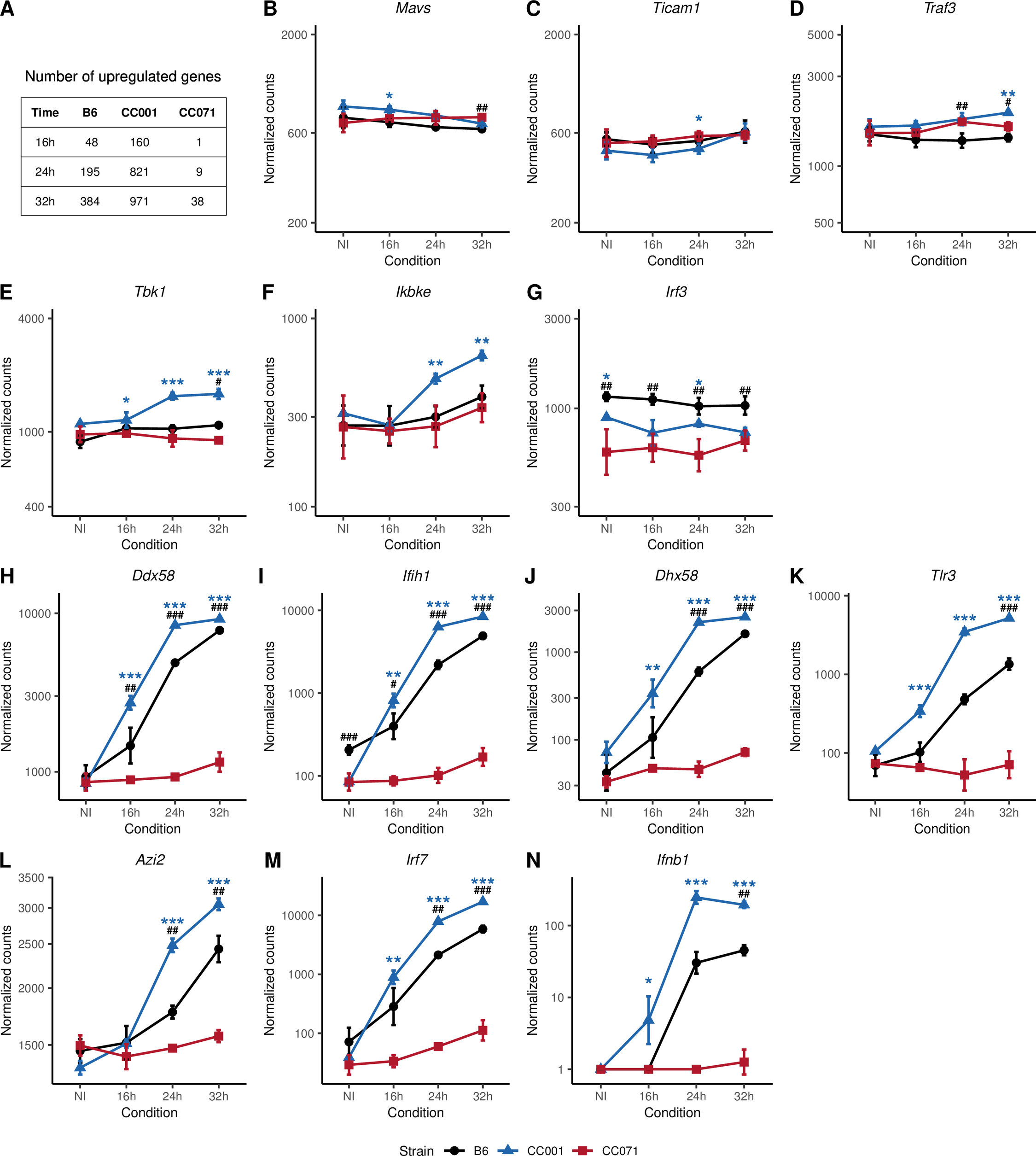

Supplement: S3 Fig — MEFs derived from B6 (black circles), CC001 (blue triangles) and CC071 (red squares) were infected with ZIKV at a MOI of 5. mRNA expression levels were measured by RNAseq in non-infected (NI) and ZIKV-infected MEFs. (A) Number of upregulated genes per strain at 16, 24 and 32 hpi (log2 fold-change > 1, FDR = 0.05). (B-G) Genes constitutively expressed. (H-N) Genes which expression is induced by the IFN-I response (ISGs). Expression levels are shown on a logarithmic scale. For Ifnb1 expression, null counts were transformed to 1. Data are mean +/- sem from 3 biological replicates. Blue asterisks and black hashes show statistical significance of CC071 compared to CC001 and to B6, respectively (ANOVA followed by post-hoc Tukey HSD, */# p < 0.05, **/## p < 0.01, ***/### p < 0.001). (TIF) [file ppat.1011446.s003.tif]

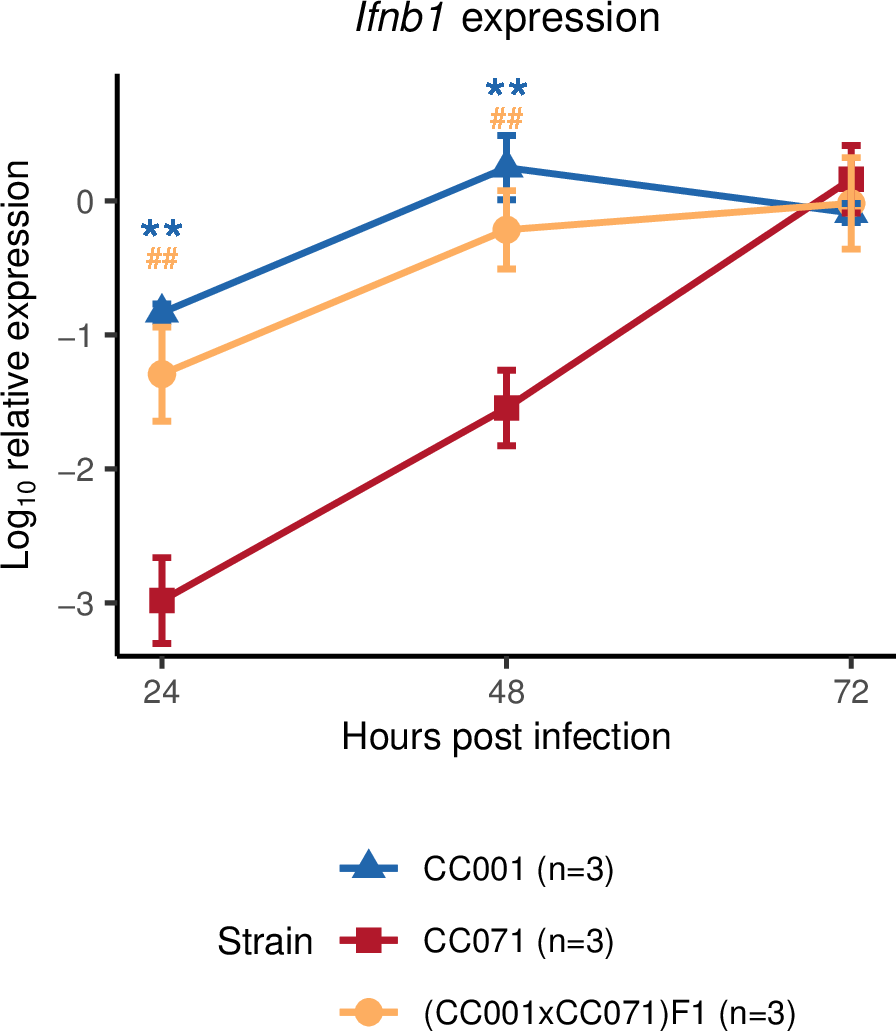

Supplement: S4 Fig — Ifnb1 expression upon ZIKV infection in CC001, CC071 and (CC001xCC071)F1 MEFs determined as in Fig 1B. Data are mean +/- sem from 3 biological replicates. Blue asterisks and orange hashes show statistical significance of CC071 compared to CC001 and to F1, respectively (ANOVA followed by post-hoc Tukey HSD, **/## p < 0.01). (TIF) [file ppat.1011446.s004.tif]

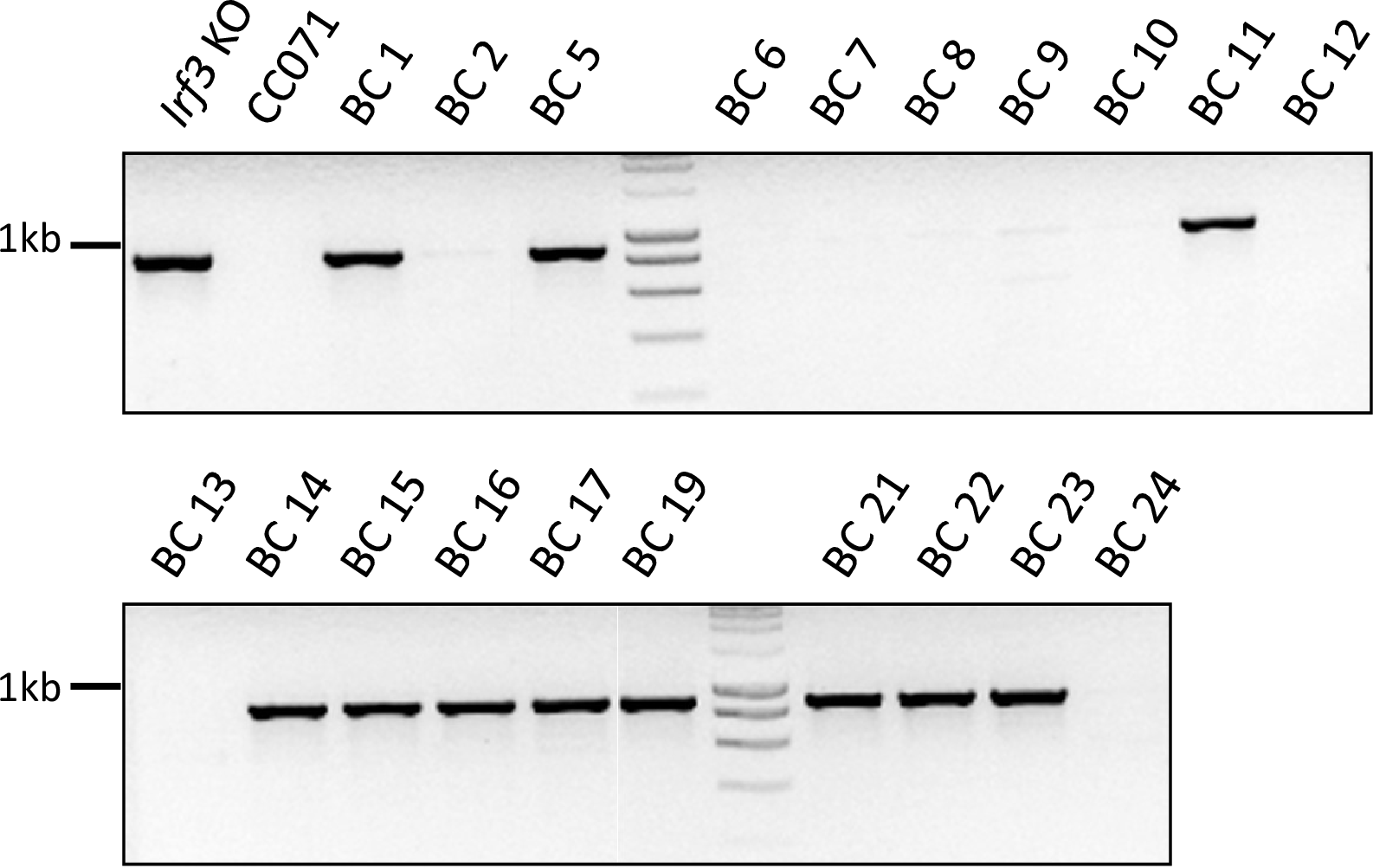

Supplement: S5 Fig — Backcross individuals were genotyped by PCR with primers amplifying the intro 6 of Irf3 (sequences in S1 Table). The Irf3KO allele results in a band at 877pb while the Irf371 allele results in no band. Individuals 1, 5, 11, 14, 15, 16, 17, 19, 21, 22 and 23 are Irf3KO/71 and individuals 2, 6, 7, 8, 9, 10, 12, 13 and 24 are Irf371/71. (TIF) [file ppat.1011446.s005.tif]
