# Supplementary material for: Susceptibility to Zika virus in a Collaborative Cross mouse strain is induced by Irf3 deficiency in vitro but requires other variants in vivo
Source: PLoS Pathog. 2023 Sep 21;19(9):e1011446. doi: 10.1371/journal.ppat.1011446 (PMC10547207; doi:10.1371/journal.ppat.1011446)
Supplement: S1 Table — (PDF) [file ppat.1011446.s006.pdf]

**Gene**	**Forward**	**Reverse**
qPCR		
Ifnb1	CAGCTCCAAGAAAGGACGAAC	GGCAGTGTAACTCTTCTGCAT
Ifna4	TGATGAGCTACTACTGGTCAGC	GATCTCTTAGCACAAGGATGGC
ZIKA *Env*	CCGCTGCCCAACACAAG	CCACTAACGTTCTTTTGCAGACAT
Ifitm3	ACTGTGATCAACATGCCCAGAG	CTTCCGATCCCTAGACTTCACG
Tbp	AGAACAATCCAGACTAGCAGC	GGGAACTTCACATCACAGCTC
PCR genotyping		
Irf3	CACTCCCACGCCTTCTGG	ACAAGCCTCTTGACCCATGG
