# Supplementary material for: Susceptibility to Zika virus in a Collaborative Cross mouse strain is induced by Irf3 deficiency in vitro but requires other variants in vivo
Source: PLoS Pathog. 2023 Sep 21;19(9):e1011446. doi: 10.1371/journal.ppat.1011446 (PMC10547207; doi:10.1371/journal.ppat.1011446)
Supplement: S2 Table — (PDF) [file ppat.1011446.s007.pdf]

Immunofluorescence antibodies

Primary/secondary	Antibody	Species	Dilution	Reference
Primary	ZIKV envelop protein	Mouse	1:200	4G2 antibody from ATCC hybridoma
Primary	Phosphorylated IRF3	Rabbit	1:250	Cell Signaling Technology Cat#29047
Secondary	Anti-rabbit AlexaFluor-488-conjugated IgG	Goat	1:500	Invitrogen Cat#A-11034
Secondary	Anti-mouse AlexaFluor-594-conjugated IgG	Donkey	1:500	Invitrogen Cat#A-21203

Western blot antibodies

Primary/secondary	Antibody	Species	Dilution	Reference
Primary	IRF3 Cter	Rabbit	1:1000	Cell Signaling Cat#4302
Primary	Vinculin	Rabbit	1:1000	Proteintech Cat# 26520-1-AP
Secondary	Rabbit IgG HRP Linked Whole Ab	Donkey	1:10,000	Sigma Cat# GENA934-100UL
